# Integron and its role in antimicrobial resistance: A literature review on some bacterial pathogens

**DOI:** 10.22038/ijbms.2020.48905.11208

**Published:** 2021-02

**Authors:** Parisa Sabbagh, Mehdi Rajabnia, Amirhosein Maali, Elaheh Ferdosi-Shahandashti

**Affiliations:** 1 Infectious Diseases and Tropical Medicine Research Center, Health Research Institute, Babol University of Medical Sciences, Babol, Iran; 2 Department of Microbiology, Faculty of Medicine, Babol University of Medical Sciences, Babol, Iran; 3 Student Research Committee, Pasteur Institute of Iran, Tehran, Iran; 4 Department of Medical Biotechnology, Faculty of Allied Medicine, Qazvin University of Medical Sciences, Qazvin, Iran; 5 Department of Medical Biotechnology, Faculty of Medicine, Babol University of Medical Sciences, Babol, Iran

**Keywords:** Antibiotic resistance, Gene cassettes, Integrases, Integrons, Mobile elements

## Abstract

In recent years, different acquired resistance mechanisms, including transposons, bacteriophages, plasmids, and integrons have been identified as involved in the spread of resistance genes in bacteria. The role of integrons as mobile genetic elements playing a central role in antibiotic resistance has been well studied and documented. Integrons are the ancient structures that mediate the evolution of bacteria by acquiring, storing, disposing, and resorting to the reading frameworks in gene cassettes. The term integron describes a large family of genetic elements, all of which are able to capture gene cassettes. Integrons were classified into three important classes based on integrase *intI *gene sequence. Integrons can carry and spread the antibiotic resistance genes among bacteria and are among the most significant routes of distribution of resistance genes via horizontal transfer. All integrons have three essential core features. The first feature is *intI*, the second one is an integron-associated recombination site, *attI*, and an integron-associated promoter, Pc, is the last feature. Among them, the class 1 integron is a major player in the dissemination of antibiotic resistance genes across pathogens and commensals. Various classes of integrons possessing a wide variety of gene cassettes are distributed in bacteria throughout the world. This review thus focuses on the distribution of integrons among important bacteria.

## Introduction

Integrons are the ancient structures that mediate the evolution of bacteria by acquiring, storing, disposing, and resorting to the reading frameworks in mobile elements called cassettes. They are present in approximately 17% of the bacterial chromosomes (1). These structures are found in different environments such as forest, desert soils, river sediments, antarctic soils, hot springs, biofilms, plant surfaces, marine sediments, and deep-sea sediments. Nowadays, the term integron describes a large family of genetic elements, all of which are able to capture gene cassettes. Although about one-third of integrons have been found in bacterial genome that do not carry gene cassettes (empty integrons) (2). Although the first antibiotic-resistant bacteria were reported in the mid-1950s, until the 1970s it was not clear that resistance phenotypes were associated with transmissible plasmids or elements. In the late 1980s, integrons were identified. Integrons play an important role in the distribution of antibiotic resistance, especially in Gram-negative pathogens. In resistance integrons, an action plan is associated with genetic moving elements such as transposons or plasmids, so interspecies and intraspecies transmission are increased. It has now been well established that integrons act as the main reason for multiple resistance in Gram negative more than in Gram-positive bacteria (3).

***Classification of integrons***

Integrons can be discriminated based on the relative homology of *intI*, although the cutoff point is not clear (2). Initially, it was suggested that integrons can be divided into two categories: 1) mobile integrons: have a small number of cassettes, usually encode antibiotic resistance, have different *attC* sites and their mobility is dependent on transposons or plasmids, 2) super integrons: have many cassettes, homogenous *attC* sites and located on the chromosome (4). Also, phylogenetic studies showed that a branch of integrons is associated with organismal phylogeny. These integrons are divided into three groups based on the phylogeny of the integrase genes: (i) a group of isolated *Proteobacteria* from freshwater and soil, including clinical integrons in classes I and III. (ii) a group found in *Gamma-Proteobacteria* in marine environments that includes class II, SXT integrative conjugative elements integrons and pRSV1 plasmids in *Vibrio*; and (iii) the integrons whose integrase genes are inverted. These inverse integrons have been found in *Spirochaetes*, *Planctomycetes*, *Cyanobacteria*, and *Chlorobi *spp. (4). Up to now, more than 9 classes of integrons have been identified based on 16 amino acids conserved in Gram-negative bacteria (5), but only 4 main classes are associated with clinical isolates. As stated above, the amino acid sequencing of the integrase gene is used as a marker for the classification of integrons into different classes, and members having the same integrase have the same class but can carry different gene cassettes. Of all the different classes of integrons, the class I integrons and then class II are the most common classes among clinical isolates (6). Classes I to III are also integrons with multiple resistance. The class IV integrons are considered a distinct type of integron called super integron, which is found on the small chromosome *Vibrio cholera *(7).

***Integrons structure***

**Figure1:**
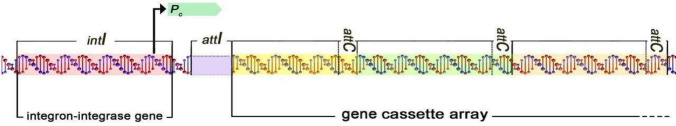
Integron structure intI, a gene for the integron integrase; attI, recombination site; Pc, an integron promoter. Gene cassettes, sequentially inserted into an array via recombination between attI and the cassette associated recombination sites (attC)

Structurally, all integrons consist of three main components, including 5′ and 3’ conserved segment and a central variable region between the 5’ and 3’ zone, in which integrons are responsible for the capture and expression of exogenous genes, which are part of the gene cassette (1). Essential components of the 5’ and 3’ zone in all integrons include 1) integrase gene (*intI*) encoding a specific recombinant site and a part of the family of tyrosine recombinase (8), 2) the *attI* receptor site identified by integrase and adjacent to the *intI* gene, so that *attI* is in the upstream of *intI*, except for the *Triponama* spp. (9). Also, integrase protein can catalyse a recombinant between the input gene cassette and the *attI*. 3) the promoter sequence consists of *P*_c _and *P*_int_ which is placed inside *intI* or between *intI* and *attI* and causes expression of the existing genes in the integrated gene cassette in integrons and integrase (9). The 3’ conserved segment of integrons has different structures that differ in integron classes. The gene cassettes are located between the 3’ and 5’ zone, where integrons receive new genes through these cassettes. The integron system has two important benefits as a genetic innovation: the new genetic material is integrated into the bacteria genome at *attI*, therefore, they do not cause abnormalities in the existing genes; second, the new integrated genes are expressed by the integrons promoter (*Pc*) (Figure 1) (10). 

***Gene cassette***

**Figure 2:**
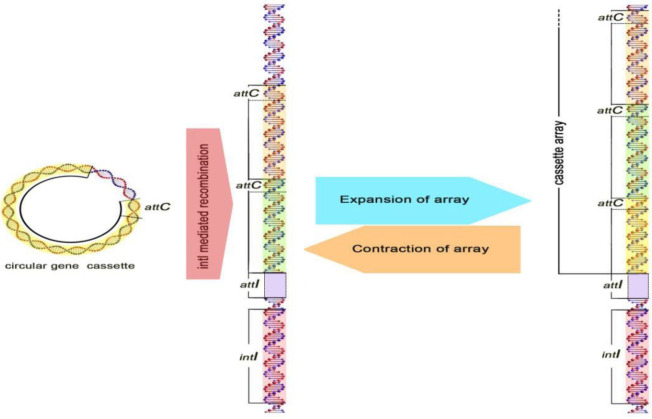
Acquisition of gene cassettes. Integrons acquire new gene cassettes by recombination between attC of a circular cassette and the attI site of integrons

The number of cassettes reported differ from zero to 100 in different studies, this difference in the number of cassettes is seen in *Vibrio cholerae* and some other species of *Vibrio*. A great variety is created in integron classes through the acquisition of various gene cassettes (11). Many studies have been done on the origin of the gene cassettes, but so far with no result. However, the size and frequency of gene cassettes suggest that some organisms, using single or pair genes, can create a gene cassette, the mechanism of which method has not yet been identified. These cassettes are mobile elements that code multiple genes, including antibiotic resistance genes. These antibiotic classes include all known β-lactams, all aminoglycosides, chloramphenicol, trimethoprim, streptothricin, erythromycin, quinolones, rifampin, lincomycin, fosfomycin, and antiseptics of the quaternary ammonium-compound family (1, 3). According to previous studies, gene cassettes are randomly combined in the region between the 3’ and 5’ conserved segment of integrons (12). The integration of gene cassettes is done with the integrase gene between two recombination sites (*attI*, *attC*) which process is reversible and cassettes can be released in the form of free DNA from integrons (Figure 2) (11). In general, gene cassettes are compact and dense DNA that have a simple structure, consisting of two components of the recombination site and the open reading frame (ORF) (13). Gene cassettes lack the promoter sequences in their structure, thus expressing the gene cassettes associated with an external promoter (*Pc*) in the structure of integrons. Because integrons have promoter sequences, they can express the genes in the gene cassettes, so integrons act as both the expression vector of the gene and as a natural cloning system (12). There are areas at the end and beginning of a gene cassette that consist of a protected sequence including GTTRRRY (representing recombination activity). Although *attC* has distinct sizes between 57-141 bp, they have a set of common characteristics. These elements have a core site (CS) with the sequence GTTRRRY and an inverse core site (ICS) with the sequence RYYYAAC. The *attC* sequence was formerly known as 59bp element (14). All 59bp elements have a symmetrical central axis in their structure, on both sides of this axis of symmetry, there are ICS and CS sites, which has led to the creation of hairpins in the 59bp element. The attC sequences are categorized into different groups, based on the size of the first and largest group of 12 members and dependent on *aacA*(*Iia*), *aadA*, *aadB*, *CatB3*, and *orfD* genes. The factors present in these genes are only different in some base pairs (15).

***Mobility***

The mobility of integrons has been considered to be a major concern in clinical pathogens of spreading antibiotic resistance, and this mobility is related to mobile DNA elements (transposon and plasmid) (16). However, integrons do not have the ability to move, but integrons (mostly Class I) are known as mobile genetic elements that are most commonly found on transferable plasmids. Therefore, these moving plasmids carry gene cassettes that can transfer to other integrons or even to the bacterial genome. The integron system allows microorganisms to combine gene cassettes and convert them into functional proteins by expressing the genes correctly (2). Mobile genetic elements containing plasmids, transposons, secretion sequences, and genetic islands can act as extensive reservoirs of information for integrons, which are shared among bacteria. With the motion of the gene cassettes, the integrons play an important role in the distribution and spread of resistance genes. In addition to clinical aspects, there are many reports of the presence of integrons in environmental microorganisms, which indicate their high diversity in various functions. This explains exactly that integrons are old genetic elements within the genome and play an important role in evolution and adaptation (13).

***Class I integron***

**Figure 3:**
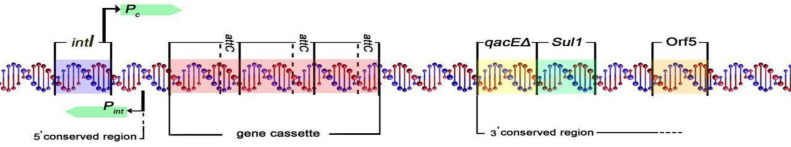
Structure of class I integron. The following cassettes are a part of the 3′conserved region and not mobile: sul1 gene encoding resistant to sulfonamides, qacEΔ encoding resistant to quaternary ammonium compounds, and orf5 unknown function

In 1998, class I integron was found for the first time in *Corynebacterium glutamicum*, which is a Gram-positive microorganism (7), first discovered by Hall and Stoke in 1989 (17), and then it was observed in *Corynebacterium, Streptococcus, Enterococcus, Staphylococcus, Aerococcus and Brevibacterium*. Class I integron has the highest frequency among the integrons (11, 18). *IntI* is able to identify three types of recombinant sites (*attI*, *attC*, and secondary sites). Therefore, these types of integrons are able to receive gene cassettes through the recombination of dedicated sites. Also, class I integron has a direct relationship with Tn402 and the Tn3 family of transposons (1, 18). In this class of integrons, the gene cassettes can be further expressed through the promoters *P*_c _and*P*_2_ (second promoter that is usually inactive). *P*_c_ plays an important role in integron function because it ensures the correct expression of the gene cassettes. Class I integron has been studied in different microorganisms. The prevalence of class I integron is about 22 to 55%, among the Gram-negative bacteria isolated from the clinic, which include *Acinetobacter, Aeromonas, Alcaligenes, Burkholderia, Campylobacter, Citrobacter, Enterobacter, Escherichia, Klebsiella, Mycobacterium, Providencia, Pseudomonas, Salmonella, Serratia, Shigella, Stenotrophomonas, and Vibrio* (19), and it is also found in about 22 to 59% of *Campylobacter jejuni, Providencia stuartii, Serratia marcescens, Stenotrophomonas maltophilia *(7). Class I integron acts as a common factor in the distribution and spread of antimicrobial resistance. This class carries over 40 resistance genes related to aminoglycosides, beta-lactams, chloramphenicol, macrolides, sulfonamides, disinfectants, and dexophthane (20). The 3′-conserved segment in class I integron consists of the following components: 1) *qacEΔ1* gene encoding resistance to quaternary ammonium salts and dexophthane, 2) *sul1* gene encoding resistance to sulfonamides (14), 3) *orf5* has no known function but is similar to puromycin acetyltransferase in *Streptomyces albonier*, this suggests that it leads to resistance to puromycin through the mechanism of acetyltransferase (Figure 3) (21). 

***Class II integron***

Class II integrons indicate a high prevalence in clinical isolates in Gram-negative bacteria such as A*cinetobacter, Shigella, Salmonella, Pseudomonas *(7). Class II integrons, similar to class I, are also associated with the Tn7 family of transposons (Tn7 and its derivatives such as Tn1825, Tn1826, and Tn4132) which carry the recombinant site *attI2* and *P*_c_. The 3′-conserved segment includes 5 *tns* (*tnsA*, *tnsB*, *tnsC*, *tnsD*, and *tnsD*) genes that play a role in the transposon movement. Class II integrons contain gene cassettes including *dfrA1* (dihydrofolate reductase), *sat1* (streptothricin-acetyl transferase), and *aadA1* (aminoglycoside adenyltransferase) which are resistant to trimethoprim, streptomycin, and streptomycin/spectinomycin, respectively. The *ereA* gene (erythromycin esterase) has also been found in the class II integron (7, 16). The integrase gene in class II integrons is about 46% similar to the integrase gene in class I integrons. One of the most important differences between *intI*1 and *intI*2 is that the integrase gene in the class II integrons (*intI*2) is stopped early by the end codons (*TAA*), resulting in the 178 amino acid protein synthesis being deactivated. Therefore, class II integrons are weaker in moving gene cassettes than class I integrons. However, mutations in end codons lead to reactivity of these amino acids, which results in the activation of the integrase gene (3).

***Class III integron***

In 1993, class III integrons were first identified in Japan by Arakawa and colleagues in *S. marcescens*. These types of integrons rarely present in clinical specimens, they have been found in a small number of bacteria such as *Acinetobacter *spp*., Alcaligenes, Citrobacter freundii, Escherichia coli, Klebsiella pneumoniae, Pseudomonas aeruginosa, Pseudomonas putida, Salmonella *spp*. and S. marcescens *(7). The 3′-conserved segment in these integrons is similar to that of class I integrons: it contains four genes, *qacEΔ1*, *sul1*, *orf5*, *orf6*, and their only difference is the lack of transcription genes. In these integrons, there are several gene cassettes including *bla*_IMP-1 _and *aacA4* that encode metallo-lactamase enzymes and the aminoglycoside resistance gene (tobramycin), respectively. The *aacA4* cassette was originally found in class I integrons but the *bla*_IMP-1_ cassette was first recognized in class III integrons, however due to the extensive recombination that occurred between class I and III integrons, this gene is also found in class I integrons and has been widely reported worldwide (22).

***Class IV integron***

In fact, class IV integrons are super integrons that were first detected in *Vibriocholera*. Mazel in 1998, for the first time, called them super integron (19). These integrons are seen in microorganisms including *Vibrionaceae, Shewanella, Xanthomonas, Pseudomonas, *and other* proteobacteria*. To date, class IV integrons have been found to carry gene cassettes imparting resistance to the antibiotic’s chloramphenicol and fosfomycin (23).

***Antibiotic resistance***

In recent years, excessive consumption of antibiotics has led to increased antibiotic resistance. Genetic mutations and acquisition of resistance genes are involved in the development of resistance. One of the most important causes of the development and spread of antibiotic resistance of genes is the acquisition of resistance genes that occur through the horizontal transduction of the gene or genetic moving elements such as plasmids and transposons (24). These integrons share a collection of genetic cassettes most of which encode antibiotic resistance. In general, about 130 resistant genetic cassettes have been identified with varying patterns of codon and *attC* sites (3). Resistance integrals have several common features: they usually have motion, their cassette arrangements are short, and typically only carry antibiotic resistance genes. However, these common features in them are not inherent characteristics of the ancestors of integrons but they are created by strong selective pressure during the use of antibiotics by humans.

***Integrons in various bacteria***

*Escherichia coli*

*E. coli* is known as the head of the large family of *Enterobacteriaceae*. Based on diversity in pathogenesis and clinical symptoms, the strains of *E. coli* are divided into two intestinal and extra-intestinal. *E. coli* is one of the most important causes of gastrointestinal and urinary tract infections in humans that has been resistant to a wide range of antibiotics over the past years (25). Acquiring mobile elements, including plasmids, transposons, and integrons among Gram-negative bacteria, it plays an important role in the development of antibiotic resistance (2). Class I integron was reported in 1973 (26). Common cassettes in this class of integron result in resistance to aminoglycosides (*aadA1, aadA2, aadB, aadA5*), trimethoprim (*dfrA1, dfrA5, dfrA7, dfrA12, dfrA17, dfrB2, dfrA1-gcuC, dfrA17-aadA5, dfrA1-aadA1, dfrA12-gcuF-aadA2), *erythromycin (*ere2*) and broad-spectrum beta-lactams (ESBL) (*bla*_OXA-101_*-aac(6’)–Ib*) (27). Also, class II integron has been reported less frequently. This integron contains gene cassettes *dfrA1-sat1-aadA1*, *dfrA1-sat2-aadA1* and *estX-sat2-aadA1 *(28). Also, recently, a new class II integron has been identified (29), which includes two gene cassettes. One of them is the *dfrA14* gene. There are also reports available on the availability of Class III integrons that are commonly seen with class I and III integrons in this bacterium (30). (Table 1) in our study in 2015, we collected *E. coli* from clinical isolates of patients in the north of Iran, which showed 22% of *E. coli* isolates carried class I integron (Table 2) (31). 

***Acinetobacterbaumannii***

*A. baumannii* is recognized as an important cause of nosocomial infections that has antibiotics resistant genes including efflux pumps, class B β-lactamase (Metallo-beta-lactamase (MBL)), class C chromosomal β-lactamase (Amp C), class D β-lactamase (OXA-type carbapenemase), integrons, and associated insertion sequence (IS) elements (32). Class I integron in this bacterium has several gene cassettes (aadA2, aadB, dfrA7, *bla*_CARB-2, _*dfrA1-gauC, dfrA17-aadA5, dfrA1-aadA1*, *dfrA12-gcuF-aadA2*) that caused resistance to aminoglycosides, ESBLs, and trimethoprim (27). Although class II integrons including* dfrA1, aadA1*, *sat1*, and *aadB* which are resistanct to trimethoprim, streptomycin, tobramycin, and kanamycin, respectively (Table 1)(33). In other studies, in 2017, we collected *A. baumannii* isolates from BAL samples of patients admitted to the ICU at Ayatollah Rouhani Hospital in Babol, Iran. The distribution analysis of *intI* genes showed that 25.7%, 88.6%, and 28.6% of isolates carried the *intI1*, *intI2*, and *intI3*genes, respectively. Also, the prevalence of *aadB*, *dfrA1*, *bla*_OXA30_, *aadA1* and bla_PSE1_ gene cassettes were 94.3%, 77.15, 40%,5.7%, and 0%, respectively (33, 34).

*Salmonella spp.*

*Salmonella *spp*.* are intestinal pathogens that are usually transmitted by contaminated food, especially animals such as meat, poultry, eggs, and milk. Multidrug-resistant (MDR) salmonella has been a major public health issue since 1990. *Salmonella *spp*. *is associated with different classes of integrons, usually containing one to three gene cassettes (35). Class I integrons play a major role in antibiotic resistance in *Salmonella *spp. This class has several types of gene cassettes resistant to aminoglycosides (*aadA*, *aadA1*, *aadA2*, *aadA5*, *aadB*), beta-lactams (bla_CARB-2_) and trimethoprim (*dfrA1*, *dfrA7*, *dfrA12*, *dfrA17*, *dfrA1*-*gcuC*, *dfrA1*-*aadA1*, *dfrA17*-*aadA5*, *dfrA12*-*gcuF*- *aadA2*) (27). Although the percentage of class II integrons is relatively lower in this bacterium, it appears in different serotypes of *salmonella *spp*.* with *dfrA1*-*sat1*-*aadA1* and *estX-sat2-aadA1* cassettes. Class III integrons are not yet found in this bacterium (Table 1)(36). In our study in 2015, the prevalence of Class I integrons in *Salmonella infantis* was 36% (Table 2) (37).

*Klebsiella spp.*

*Klebsiella *spp*. *causes diseases such as pneumonia, meningitis, and blood and urinary tract infections. This bacterium is resistant to a wide range of antibiotics (aminoglycosides, cephalosporins, and ESBLs). Class I integrons in *Klebsiella *spp*. *has different gene cassettes, including *aadA*1a, *aadA2, dfrA*7, *aadB*, *dfrA1*-*gcuC*, *dfrA1*-*aadA1*, *dfrA17*-*aadA5*, *dfrA12*-*gcuF*-*aadA2,* *aadA*, and *bla*_CARB-2_. However, multiple resistance to these bacteria has been determined due to *dfrA12-orfF-aadA2,* and *dfrA1-orfC*(27). Antibiotic resistance in *Klebsiella *spp*. *Is known more to ESBLs such as *TEM*, *SHV*, *CTX-M*. Class I integrons in *Klebsiella* spp. also has genes associated with MBLs like Verona integrin (VIM) and IMP-type carbapenemases (IMP). These MBLs hydrolyze β-lactams including carbapenems, which are commonly enclosed in class I integrons. The class II integron also has *dfrA1, sat1 / sat2, and aadA1* cassettes, and class III integron includes *bla*_GES-1 _(Table 1) (38). Also, we studied *K. pneumonia *isolates in 2011 and 2015 . Our results showed that 36.6% of isolates carried the *intI1 *gene and the prevalence of *bla*_VIM-1 _gene cassette was 30% (Table 2) (39, 40).

*Pseudomonas aeruginosa*

*P. aeruginosa* is one of the most important factors in hospital infections, especially in burn patients and patients with cystic fibrosis. Due to increased MDR bacteria, treatment for this bacterium is difficult to find. Class I integron is an important factor for the development of antibiotic resistance and the emergence of MDR strains (41). This class of integrons includes some gene cassettes such a *aadB* and *aadA*2 (resistance to aminoglycosides), *dfrA17-aadA5* and *dfrA12-gcuF-aadA2* (resistance to trimethoprim), bla_CARB-2 _(resistance to carbenicillin) (27). Class II integrons in *P. aeruginosa* include *dfrA1-sat1-aadA1 *(Table 1)(16). Our studies in 2012 and 2013 on *P. aeruginosa* isolates showed 37–40% of isolates had the* intI1 *gene (Table 2) (41, 42).

*Enterococcus faecalis*

*E. faecalis* is an intestinal flora and has been identified as one of the major causes of hospital infections, with a tendency to increase antibiotic resistant class I integrons; *E. faecalis* contains gene cassettes *dfrA12-gcuF-aadA2* (resistance to trimethoprim) and *aadA1a *(resistance to aminoglycosides)(43). Class II integrons has the *dfrA1-sat1-aadA1 *cassettes (Table 1) (44)***.***

*Enterobacter spp.*

*Enterobacter *spp*.* are Gram-negative bacteria that cause gastrointestinal diseases, at present, *Enterobacter *spp*.* with MDR patterns that contain class I integron are considered one of the major concerns of physicians and infection control practitioners. This class causes resistance to aminoglycosides (*dfrA7, aadA1a, *and* aadA2*) and trimethoprim (*dfrA1-aadA1, dfrA17-aadA5, dfrA12-gcuF-aadA2*, and *dfr12-orfF-aadA2*)(Table 1)(27)*.*

*Staphylococcus aureus*

*S. aureus* is a Gram-positive microorganism which is now considered one of the most important causes of hospital infections (45). Quantitative studies have been done on integrons in Gram-positive bacteria. Class I integrons is more common in *S. aureus* and causes resistance to aminoglycosides (*aadA1a* and *aadA2*), trimethoprim (*dfrA17-aadA5 and dfrA12-gcuF-aadA2*) and chloramphenicol (*aacA4*-*cmlA1*)( Table 1) (46)***.***

**Table 1:** Common gene cassette arrays in types of integrons in diverse bacterial species and their role in antimicrobial resistance

References	Antibiotics associated with gene cassettes	gene cassettes	Integrons	Bacteria
(28, 30)	Aminoglycosides, Trimethoprim, Extended Spectrum Beta-Lactamase, Erythromycin.	aadA1, aadA2, aadA5 aadB, dfrA1, dfrA5, dfrA7 dfrA12, dfr14, dfrA17, dfrB2, dfrA1-gcuC, dfrA1-aadA1, dfr17-aadA5, dfr12-gcuF-aadA2, dfrA1-sat1-aadA1, dfrA1-sat2-aadA1, estX-sat2-aadA1, bla_OXA-101_-aac (6')–Ib, ere2.	I, II, III	*Escherichia coli*
(27, 32, 33)	Extended Spectrum Beta-Lactamase, Aminoglycosides, Trimethoprim.	bla_CARB-2_, aadA1, aadA2, aadB, dfrA1, dfrA7, dfrA1-gcuF, dfrA1- aadA1, dfr17-aadA5, dfr12-gcuF-aadA2, sat1.	I, II	*Acinetobacterbaumannii*
(27, 36)	Aminoglycosides, Trimethoprim, Extended Spectrum Beta-Lactamase.	aadA, aadA1a, aadA2, aadA5, aadB, dfrA1, dfrA7, dfrA12, dfrA17, dfrA1-gcuF, dfrA1-aadA1a, dfr17-aadA5, dfr12-gcuF-aadA2, bla_CARB-2._	I, II	*Salmonella spp. *
(4, 27)	Extended Spectrum Beta-Lactamase, Trimethoprim, Aminoglycosides,	bla_CARB-2_ bla_GES-1_, aadA, aadA1, aadB, dfrA1, dfrA7, dfrA1-gcuF, dfrA1-aadA1a, dfr17-aadA5, dfr12-gcuF-aadA2.	I, II, III	*Klebsiella spp.*
(16)	Aminoglycosides, Trimethoprim	aadA2, aadB, dfr17-aadA5, dfr12-gcuF-aadA2.	I	*Pseudomonas aeruginosa*
(46)	Aminoglycosides, Trimethoprim, Chloramphenicol	aadA1, aadA2, dfr17-aadA5, dfr12-gcuF-aadA2, aacA4-cmlA1	I	*Staphylococcus aureus*
(43)	Aminoglycosides, Trimethoprim	aadA1a, dfr12-gcuF-aadA2, dfrA1-sat1-aadA1.	I	*Enterococcus faecalis*
(44)	Aminoglycosides, Trimethoprim	aadA1a, aadA2, dfrA7, dfrA1-aadA1a, dfr17-aadA5, dfr12-gcuF-aadA	I	*Enterobacter spp.*

**Table 2:** Prevalence of different types of integron and their gene cassettes in our studies between 2011-2017

Bacteria	Class I integron	Class II integron	Class III integron	Frequency of gene cassettes	References
*Escherichia coli*	22%	---	---	---	(31)
*Acinetobacterbaumannii*	25.7%	88.6%	28.6%	aadA1(5.7), bla_OXA30_(40%), aadB(94.3%), dfrA1(77.1%),	(33, 34)
*Salmonella infantis*	36%	---	---	---	(37)
*Klebsiellapneumoniae*	36.6%	---	---	Bla_VIM-1_(30%)	(39, 40)
*Pseudomonas aeruginosa*	37–39.5%	---	---	---	(41, 42)

## Conclusion

Integrons being capable of integrating, expressing, and disseminating gene cassettes, carrying resistance determinants, play a critical role in facilitating the MDR phenotype in these bacteria. The ability of integrons to acquire new cassettes and their ability to recombine cassette rows emphasizes the adaptation of their diversity in bacteria. Their ability to rapidly spread resistance phenotypes makes it important to consider what other integron-mediated traits, such as increased pathogenicity, virulence, or resistance to antimicrobials might impact human health in the future. If we can control integrons and cassette formation, we could use integrons as a platform for enzyme discovery and to construct novel biochemical pathways in antimicrobial resistance. So, knowledge about the prevalence of integrons and gene cassettes is helpful for the treatment and correct use of antibiotics. 
